# Rethinking Sterile: The Hospital Microbiome

**DOI:** 10.1289/ehp.122-A182

**Published:** 2014-07-01

**Authors:** Carrie Arnold

**Affiliations:** Carrie Arnold is a freelance science writer living in Virginia. Her work has appeared in *Scientific American*, *Discover*, *New Scientist*, *Smithsonian*, and more.

**Figure:**
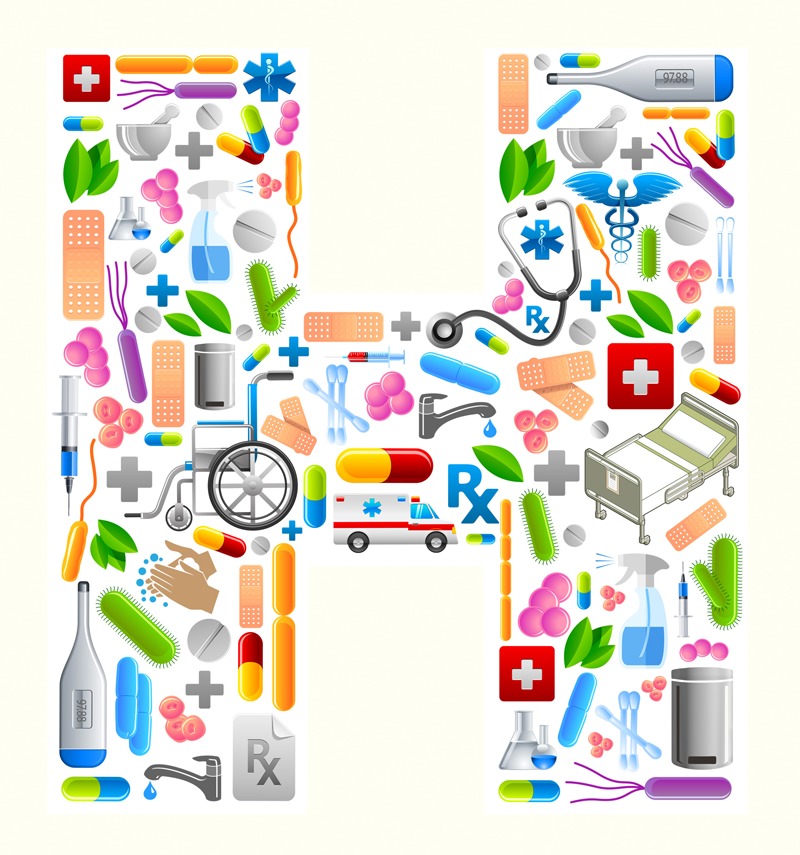
Researchers are beginning to look at hospitals as ecosystems unto themselves. “Scientists want to study the ecosystem of the hospital to understand which microbes show up where,” says microbial ecologist Jonathan Eisen. © exdez/iStock; Jane Whitney; Joseph Tart

When the University of Chicago’s new hospital pavilion opened in February 2013, it looked pristine. Floors shone, and stainless steel gurneys gleamed in the new Center for Care and Discovery. Even after the doors opened and the first patients were admitted, surfaces still looked largely sterile. It was exactly as it seemed a hospital should be: as devoid of microbial life as humans could possibly make it.

Jack Gilbert’s data told a different story. Gilbert, an environmental microbiologist at Argonne National Laboratory, and his platoon of graduate students, postdocs, and research assistants descended on the hospital several times each day, even before it opened to the public. Armed with cotton swabs, they focused their efforts on the floors devoted to surgery and oncology. Each team member took samples from floors, beds, linens, sinks, computers, nurses’ stations, air vents, and more. If you could name it, Gilbert’s team rubbed it with a cotton swab to obtain a small sample of the microbes living there.

They repeated this process several times a day for more than a year as part of the Hospital Microbiome Project, an $850,000 endeavor funded by the Alfred P. Sloan Foundation to learn more about the microbial community, or microbiome, in various hospital environments—how microorganisms transfer between humans and surfaces, and how the microbiomes develop over time. The researchers believe they can potentially reduce hospital-acquired infections by understanding the array of microorganisms that live in hospital environments, identifying the operational characteristics of buildings that influence these microbiomes, and tweaking indoor ecosystems to help prevent the spread of pathogens. A future portion of the study will involve in-depth analysis of the microbiome of a single room at an Army hospital in Germany over 16 months.

“When a pathogen invades, it doesn’t do this in isolation; it does this in the context of thousands of other species,” Gilbert says. “Very few studies have examined the rest of the communities that exist in hospitals.”

A few researchers are beginning to look at hospitals as ecosystems unto themselves. “Scientists want to study the ecosystem of the hospital to understand which microbes show up where,” says Jonathan Eisen, a microbial ecologist at the University of California, Davis.

## Hospital-Acquired Infections

In 2010 (the latest year for which data are available) 35.1 million Americans spent at least one night in a hospital.^1^ The Centers for Disease Control and Prevention estimates that 5% of patients admitted to hospitals will acquire an infection during their stay, potentially leading to 99,000 deaths annually^2^ and costing $10 billion per year.^3^

Hospital-acquired infections aren’t a new phenomenon. As long as sick people have sought care in hospitals, there has been the potential for the spread of infectious disease. With the advent of penicillin and other antibiotics, concerns about disease transmission diminished because physicians believed they had a magic bullet to fight whatever infections a person might acquire.^4^ The rise of antibiotic-resistant bacteria has changed that thinking.

Today, antibiotic-resistant infections show no signs of stopping, nor do hospital-acquired diseases.^3^Historically, these infections have been blamed on the presence of harmful bacteria, and increasingly stringent infection-control procedures and standards for sterility have been seen as the solution.^5^ A new hypothesis says that hospital-acquired infections are being driven not by the existence of harmful microbes but by the absence of helpful species.

Underneath the bright lights and on the stainless steel gurneys lives a large community of microorganisms, most of which are harmless and some potentially beneficial.^6^ Hospital microbiomes, some researchers think, form a key part of a hospital’s “immune system” and in some cases may help protect patients against infectious diseases.

“For the past 150 years, we’ve been literally trying to just kill bacteria. There is now a multitude of evidence to suggest that this kill-all approach isn’t working,” Gilbert says. “We’re now trying to understand that maybe, just maybe, if we could cultivate nonpathogenic bacteria on hospital surfaces, then we could see if that would lead to a healthier hospital environment.”

Human microbiome research has shown that the use of antibiotics can disrupt the normal array of microbes that live in and on our bodies.^7^ The constant attempts at sterilization in hospitals might function on a similar level—the use of broad-spectrum antibiotics, bleach, and hand sanitizer might take out some of the harmful pathogens, but it also cuts a swath through the hordes of nonpathogenic microorganisms.

The elimination of these commensal microbes reduces competition, potentially making hospitals more friendly toward pathogenic species, Eisen notes. “Some sterilization efforts may not be helpful in the long run because you’re going to be clearing out ecosystems which are then vulnerable to being recolonized by pathogens and not just regular, boring bacteria,” he says.

“The vast majority of these microbes are barely surviving,” says microbial ecologist James Meadow, a postdoc at the Biology and the Built Environment Center at the University of Oregon. “The built environment appears to be more of a waiting room for these potentially harmful bacteria until better conditions are present. Very few are actually enjoying themselves.” The elimination of other microbes might take the destructive pathogens out of the waiting room and into action.

## Rethinking Sterile

Historically, microbiologists have studied bacteria in pure culture, growing one species at a time. Although this allowed them to create large numbers of microbes relatively quickly, the method had several limitations.

Many microbes found in the environment are difficult, if not impossible, to grow in culture,^8^ which caused microbiologists to dramatically underestimate the number of microbes that make up human microbiomes.^9^ As well, given the significant public health threat of infectious diseases, researchers preferentially focused their efforts on harmful bacteria rather than neutral or beneficial species.^10^

**Figure:**
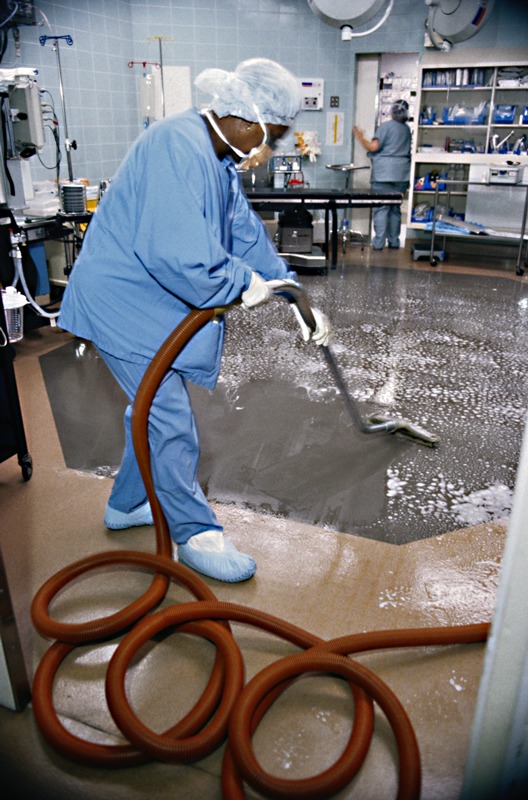
The traditional approach to hospital hygiene has been to sterilize surfaces as much as possible, but investigator Jack Gilbert says, “There is now a multitude of evidence to suggest that this kill-all approach isn’t working.” © Keith Brofsky/Getty

As recently as 50 years ago, when much of what scientists knew of microbes was related to pathogens, stripping surfaces of their thin layer of microbes made sense. But that’s not the case today. Beginning in the late 1990s and early 2000s, genetic sequencing technology began a series of exponential improvements,^11^ which lowered both the time and the cost to sequence genes.^12^ This allowed Eisen, Gilbert, and others to improve upon methods pioneered by microbial ecologist Norman R. Pace in the 1980s to survey the environment for microorganisms that cannot be grown in the laboratory; these studies showed that our world is awash in microbes.^13^ Surveys of the environment reveal that culturable bacteria such as *Streptococcus* and *Staphylococcus* species represent only a tiny fraction of the microbes we encounter every day.^14^

Different types of bacteria contain a unique version of the 16S rRNA gene that acts as a fingerprint. By swabbing a surface and then sequencing the various 16S rRNA genes present in that sample, scientists can quickly, cheaply, and easily discover the types of bacteria present in any given location.^15^ The biodiversity present on a table, bed rail, or patch of floor can rival that seen in any Amazonian rainforest, researchers have discovered.

“It’s very hard to clear out all of the microbes from a particular ecosystem,” Eisen says. In a review published in *Genome Biology*, Gilbert and coauthor Scott Kelley wrote that there “probably exists a microbe that will survive on almost any [built environment] surface or condition.”^16^ Simply put, sterility doesn’t exist.

## The Microbes in Our Midst

Far from being a homogeneous layer of unicellular life, scientists have discovered that the microbes in buildings vary widely depending on environmental conditions and the people who inhabit the rooms. Some members of indoor microbiomes are precisely what scientists would have expected. Past studies have shown the bacteria colonizing hospital therapy pools^17^ and showerheads^18^ to be moisture-loving, soil-dwelling *Mycobacteria* and *Proteobacteria*. A separate study found that showerheads were also populated by opportunistic potential pathogens that are significantly different from microbes found elsewhere in patient rooms. These bacteria tend to form biofilms, persistent colonies of microbes that favor wet, phosphorous-rich environments and can be next to impossible to kill.^19^

Showers aren’t the only hospital spaces that select for a unique array of microbes. When investigators at the University of Colorado Boulder surveyed the microbes present in Foley catheters—a common source of hospital-acquired infections^20^—they found that the bacteria present on the outside of the catheter were significantly different from those on the inside.^21^ In an Australian study, arterial catheters from intensive care units showed a similar pattern.^22^

Most of the microbes present in the hospital environment, however, arrive via humans, whether brought in on the soles of our shoes, on our cell phones, or our bodies themselves. Like Pigpen’s permanent aura of dirt in the “Peanuts” cartoon, humans are surrounded by a cloud of microbes.^23^ “Humans shed microbes wherever we go,” Gilbert says.

Each time we touch an object, we can (and do) transfer millions of microbes from our body to the environment.^24^ Because the types of microbes available to be transferred vary from person to person^25^ and body part to body part,^26^ different surfaces are likely to host different species. Objects such as computer keyboards, light switches, and soap dispensers are continually reseeded with microbes from our hands each time they are touched.^23^^,^^27^^,^^28^ Restrooms, on the other hand, are dominated by microorganisms associated with the gastrointestinal and urogenital tracts.^27^

**Figure:**
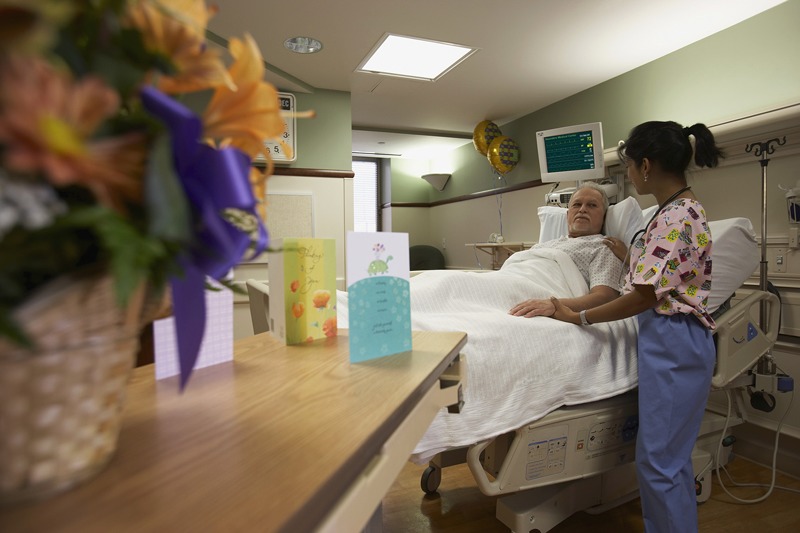
Preliminary findings indicate that the microbiome of a hospital room can change within hours to reflect the composition of the latest inhabitant. Additional new microbial species may be introduced by visitors. © Blend Image/Shutterstock

A detailed analysis of the microbiome in a classroom revealed that the types of species found varied depending on the type of human contact each sampled surface received.^29^ Chairs, the researchers found, carried a preponderance of microbes from the gastrointestinal and urogentital tracts, as well as from skin. The floors and walls were dominated by species from outdoors, likely brought in on shoes and introduced through the ventilation system.^30^

Gilbert’s preliminary, unpublished results from the Hospital Microbiome Project show that, within hours of a new patient’s arrival, the microbes in a room changed to reflect the composition of the latest inhabitant. “Within hours, the new person’s microbiome became the dominant force in that room,” he says.

The reverse process has also been demonstrated, according to research analyzing the microbes found in the neonatal intensive care unit (NICU) at Magee Womens Hospital of the University of Pittsburgh Medical Center.^31^ Just as many of the microbes found in the built environment are associated with humans, researchers now know that humans can acquire many of their microbes from their environment. Brandon Brooks, a microbial ecologist at the University of California, Berkeley, and first author of the NICU study, says that studying the indoor microbiome wasn’t just to take a microbial census. Necrotizing enterocolitis, an infection linked to multiple bacterial species, is especially deadly in low-birth-weight babies.^32^ Knowing how babies in the NICU acquired these microbes could help doctors prevent outbreaks.

Although newborns aren’t completely sterile, they have a microbiome that is much less diverse than at any other time in their lives.^33^ “We get to start with a clean slate, which makes the signals we observe different,” Brooks explains.

Brooks and colleagues collected fecal samples from the NICU newborns every three days. For each fecal sample, they also collected 33 environmental samples from around the NICU. The main species of bacteria they found in the infants’ guts (*Staphylococcus epidermidis*, *Klebsiella pneumoniae*, *Bacteroides fragilis*, and *Escherichia coli*) were found throughout the NICU, suggesting the hospital environment may have been the source of these microbes.^31^ “The next step is to try some more progressive environmental regulation strategies to see if we can cultivate an ecosystem that is beneficial for occupants,” Brooks says.

## A Breath of Fresh Air?

Sorting out the factors that influence the makeup of indoor microbial communities could help scientists identify spaces and objects at high risk of carrying pathogens. These researchers also believe simple tweaks to building design, such as altering humidity and ventilation systems, could help reduce the number of pathogens in the indoor environment. Architects are looking at such tweaks in the context not just of hospitals but also other public areas.

**Figure:**
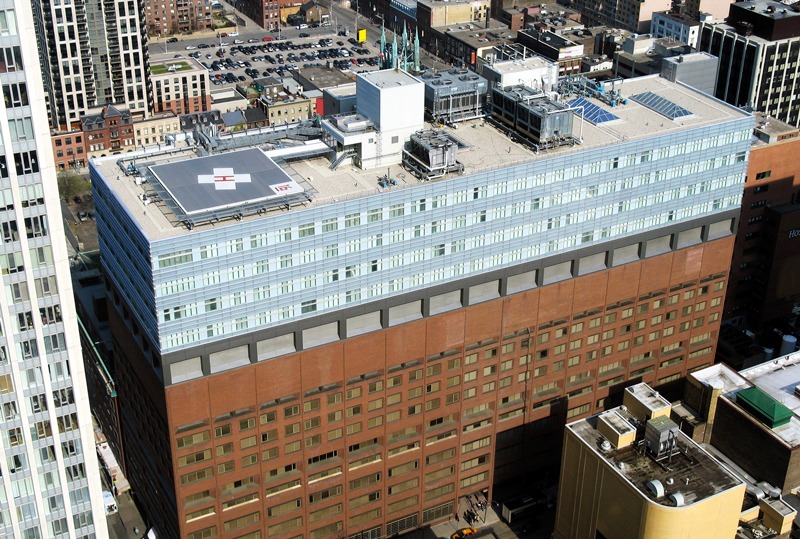
HVAC systems are a major contributor to indoor microbiomes. Some researchers believe simple tweaks to building design, such as altering HVAC systems, could help reduce the number of pathogens in the indoor environment. © Peter Spiro/iStock

The heating, ventilation, and air-conditioning (HVAC) system in a building can be a major source of indoor microorganisms, as many microbes travel through the air.^34^^,^^35^ Susannah Tringe, a microbiologist at the Department of Energy’s Joint Genome Institute, and colleagues sampled bacteria in the air-handling units of two densely populated urban shopping centers. They identified as many as 300 distinct species, many of which were both associated with the human microbiome and significantly different from those seen in outdoor air.^36^

“There’s actually a lot you can do to limit airborne transmission, and that’s where changes to the built environment can probably have the biggest impact,” says Brent Stephens, an architectural engineer at the Illinois Institute of Technology, who was not involved with the Tringe study.

**Figure:**
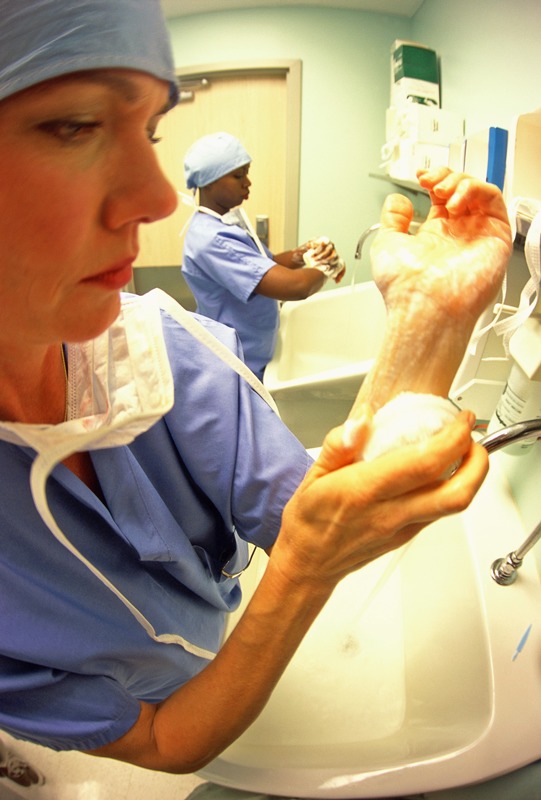
Simple behavioral changes could foster healthier hospital environments. For instance, hand washing—key to controlling the spread of many diseases—remains inadequate among hospital staff despite numerous educational campaigns. © Arthur Tilley/Getty

The architectural design of a space may affect which microbes are found there, according to research done by Meadow. Many of the microbes in indoor spaces arrived via the HVAC system,^35^ and the makeup of the microbial community varied depending on whether that system introduced outdoor air. The scientists also found that rooms in close physical proximity to one another tended to have similar microbial compositions, as did rooms with high levels of human traffic.^37^

A study of the microbiome of Oregon’s Providence Milwaukie Hospital showed that indoor air samples contained a larger percentage of bacteria related to potential pathogens than outdoor samples. Rooms that had the highest rates of airflow and humidity were associated with fewer human-associated bacteria and potential pathogens.^38^

Improving the environmental health of hospitals, however, doesn’t depend on simple scientific bean counting. Understanding how pathogens are transmitted from place to place and person to person means understanding the microbial ecosystems that live in hospitals. This understanding might not sound like a huge shift, says Meadow, but it has revolutionized how scientists think about hospitals and the microbes that live there. “If we disturb one thing by moving or sterilizing it, we need to understand what else might change,” he says.

Architects and environmental engineers alike are beginning to think about microbes when designing new hospitals and retrofitting old ones. Some of the changes may be simple, like not placing restrooms next to areas where food is prepared, to prevent the bathroom microbiome from migrating into the kitchen. Meadow hypothesizes that installing windows that can open to the outside may also help seed the hospital with a different microbial community.^39^ These environmental changes also need to happen alongside behavioral ones, Eisen says, such as improving hand washing among hospital staff, which remains subpar despite numerous campaigns.^40^

Changing the hospital environment to prevent infections seems like a new idea, borne of high-throughput genetic sequencing and other advancements of modern biology. But Meadow notes that it might just be an old idea whose time has come.^41^ “Back in the 1800s, Florence Nightingale knew that patients did better with an open window,” he says. “We’ve known for a while that just opening a window can drastically change the microbes around us in the air, and this might just influence our health in the long run.”
